# Graphene/Semiconductor Heterostructure Wireless Energy Harvester through Hot Electron Excitation

**DOI:** 10.34133/2020/3850389

**Published:** 2020-06-08

**Authors:** Yangfan Xuan, Hong Chen, Yan Chen, Haonan Zheng, Yanghua Lu, Shisheng Lin

**Affiliations:** ^1^College of Microelectronics, College of Information Science and Electronic Engineering, Zhejiang University, Hangzhou 310027, China; ^2^State Key Laboratory of Modern Optical Instrumentation, Zhejiang University, Hangzhou 310027, China

## Abstract

Recharging the batteries by wireless energy facilitates the long-term running of the batteries, which will save numerous works of battery maintenance and replacement. Thus, harvesting energy form radio frequency (RF) waves has become the most promising solution for providing the micropower needed for wireless sensor applications, especially in a widely distributed 4G/5G wireless network. However, the current research on rectenna is mainly focused on the integrated antenna coupled with metal-insulator-metal tunneling diodes. Herein, by adopting the plasmon excitation of graphene and quantum tunneling process between graphene and GaAs or GaN, we demonstrated the feasibility of harvesting energy from the 915 MHz wireless source belonging to 5G in the FR1 range (450 MHz–6 GHz) which is also known as sub-6G. The generated current and voltage can be observed continuously, with the direction defined by the built-in field between graphene and GaAs and the incident electromagnetic waves treated as the quantum energy source. Under the RF illumination, the generated current increases rapidly and the value can reach in the order of 10^−8^–10^−7^ A. The harvester can work under the multiple channel mode, harvesting energy simultaneously from different flows of wireless energy in the air. This research will open a new avenue for wireless harvesting by using the ultrafast process of quantum tunneling and unique physical properties of graphene.

## 1. Introduction

There are many wireless sensors operating for collecting the data of temperature, sound, vibration, pressure, or motions, which consist of the Internet of Things (IoTs). As powered by small batteries, those widely distributed sensors need to be charged rather than be displaced frequently. Nowadays, electromagnetic radiation generated by Wi-Fi systems of 2.4 and 5.9 GHz [1] is increasingly ubiquitous in daily life and it would be highly desirable if wearable electronics could directly translate the radiation in the Wi-Fi band into wireless charging energy. Since the millimeter-wave band for the fifth-generation mobile communication system (5G) [2] is being increasingly popularized in both the indoor and outdoor environments, providing an abundant source of radio frequency energy which always exists, it is assumed that a wireless cell which harvests the energy at much lower frequencies in the microwave spectrum (450 MHz–10 GHz) could be a solution for charging those small sensors in IoTs, which is the focus of scientists and engineers in the next years worldwide.

**Figure 1:**
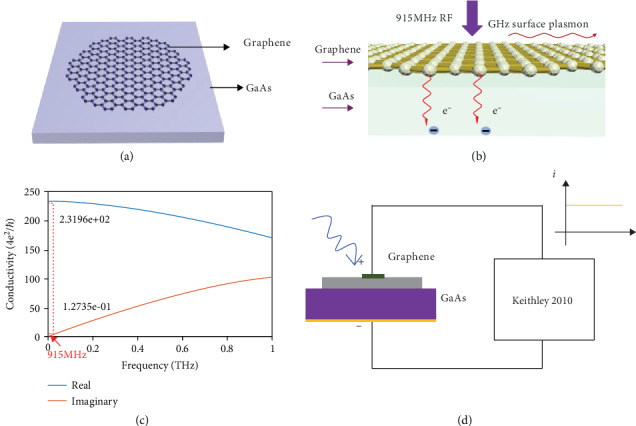
The schematic of graphene/GaAs heterojunction energy harvester. (a) The schematic structure of the device. (b) GHz plasmons in graphene. (c) Intraband conductivity of graphene at THz frequencies. (d) The schematic structure of examination on the device.

Commonly, we use a rectenna [3, 4] to harvest energy, which is a high-frequency rectifier system composed of an antenna that receives electromagnetic radiation and a metal-insulator-metal (MIM) [5] tunneling diode that converts the radiation to direct current. Recently, a flexible rectenna based on a MoS_2_ semiconducting-metallic phase heterojunction with a cutoff frequency of 10 GHz has been demonstrated [6]. Also, an optical rectenna has been realized, which directly converts freely propagating electromagnetic waves at optical frequencies into DC power [7]. However, since there is a competition between the scaled down device for high-frequency RF harvesting and the scaled up integration for high-energy absorption, a high-density integrated rectenna with an easy fabrication process remains a challenge, which is inevitable for the development of energy chips with practical wireless charging capability. Graphene may offer a key for solving this completion, since large-scale graphene can absorb all the incident electromagnetic waves and convert them into hot electrons. Innovatively, we demonstrate a large-size (in the order of cm^2^) graphene/GaAs heterostructure to harvest energy from the 915 MHz wireless source by adopting the direct excitation of graphene hot electrons and fast carrier transportation process [8] between graphene and GaAs. The device structure of the graphene/GaAs heterostructure is shown in Figure 1(a), which can be treated as a rectenna that directly converts RF energy to DC power without an external antenna. As demonstrated recently, graphene/GaAs can have a high power conversion efficiency as a solar cell, as the ~ps scale transportation process between graphene and GaAs can be very fast as a result of the high built-in electric field and narrow depletion length [9]. Besides, graphene is qualified with some excellent electrical properties, such as realizing THz SPPs [10–13], extremely high carrier mobility [14], microscale ballistic transport [15], abnormal quantum Hall effect [16], extraordinary thermal conductivity [17], and high mechanical strength [18]. With electromagnetic waves onto the graphene/GaAs heterostructure, photons are absorbed, thus generating electrons and holes with excess kinetic energy, so-called hot carriers. Because of the p-doped graphene, the Fermi level is below the Dirac point, so the electron/hole pair can be excited interband. When the photon is absorbed, the electron in the valence band absorbing the energy is excited to the conduction band and separated rapidly by the built-in electric field to form a current. The lifetime of hot electrons in graphene is normally higher than that of other quantum materials, such as semiconductor quantum dots, which allows an enough time slot for conducting the hot electrons into the GaAs substrate. Moreover, the hot electron can excite more electrons through a multiple exciton generation (MEG) process [19], which promises the effective energy usages during the cooling-down process of the hot electrons. The hot electrons and the MEG process as well as the fast transportation process between graphene and GaAs provide a great platform for wireless energy charging the sensors in IoTs.

## 2. Results and Discussion

The schematic structure of the graphene/GaAs heterojunction shown in Figure 1(a) contains silver paste, monolayer graphene, GaAs substrate, and Ti/Au. Firstly, the electrode (Ti/Au) was deposited on the back of an n-type doped GaAs substrate. Secondly, the GaAs substrate with Ti/Au was cleaned by acetone through ultrasonic cleaning for 5 minutes, followed by further washing with isopropanol solution. Then, the substrate was washed by deionized (DI) water and diluted hydrochloric acid. After that, the samples were immersed into DI water and hydrogen peroxide solution for another 5 minutes. Next, monolayer graphene initially grown on copper foil by the CVD technique was transferred to the cleaned GaAs substrate with the assistance of PMMA after etching copper foil away. After that, the sample was heated at 105°C for 15 minutes to get rid of PMMA. Finally, we added silver paste on the top of monolayer graphene as an electrode.

Figure 1(b) shows the working principle of harvesting energy from 915 MHz electromagnetic waves, which excite the plasmon in graphene as a result of collective oscillation of hot electrons in graphene. The optical conductivity  *σ* of graphene is defined as *J*/*E*, where *J* is the current density and *E* is the electric field. Generally, the overall conductivity is composed by two parts which are the intraband conductivity and the interband one and can be modeled using relaxation time approximation [20, 21]:

(1) $$\begin{array}{c} \sigma =\sigma_{\text{intra}}+\sigma_{\text{inter}},\\ \sigma_{\text{intra}}=i\frac{e^{2}k_{\text{B}}T}{\pi {\left(h/2\pi \right)}^{2}\left(\omega +i\tau^{-1}\right)}\left[\frac{\mu }{k_{\text{B}}T}+2\ln\left({\text{e}}^{-\mu /k_{\text{B}}T}+1\right)\right],\\ \sigma_{\text{inter}}=i\frac{e^{2}}{4\pi {\left(h/2\pi \right)}^{2}}\ln\left[\frac{2\left|\mu \right|-{\left(h/2\pi \right)}^{2}\left(\omega +i\tau^{-1}\right)}{2\left|\mu \right|+{\left(h/2\pi \right)}^{2}\left(\omega +i\tau^{-1}\right)}\right], \end{array}$$

where *ħ* is the reduced Plank constant, *e* is the electron charge, *k*_B_ is the Boltzmann constant, *T* is the temperature, *ω*  is the optical frequency, *μ* is the chemical potential, and *τ*_1_  and *τ*_2_ are the relaxation times of the intraband and interband scatterings. Through this model, it can be found that at the visible and infrared frequencies, the interband conductivity dominates, and graphene has a constant conductivity of  *e*^2^/4*ħ*. And in the region of 0.1-5 THz, the optical conductivity is decided by the intraband contribution. The real and imaginary parts of the conductivity of the graphene layer are shown in Figure 1(c). It has to be remarked that at this certain frequency range, graphene conductivity has a positive imaginary part but a much smaller real part, which corresponds to a negative effective permittivity with small loss. And that negative permittivity is essential for the support of SPP waves. We have to note that the phenomenological intraband scattering time, *τ*_1_ , has accounted for all scattering mechanisms including carrier-carrier scattering impurity scattering and phonon scattering [21]. Herein, we have not considered the complicated dependence of *τ*_1_  on parameters such as temperature, impurity density, and carrier density. Take *τ*_1_ = 0.6 ps, which corresponds to a mobility of 30,000 cm^2^/Vs for *μ* = 0.2 eV (carrier density 3 × 10^12^cm^2^) [22]. A typical plot of the intraband conductivity versus frequency for *μ* = 0.2 eV, *τ*_1_ = 0.6 ps, and *T* = 300 K is shown in Figure 1(c). At the frequency of 0.001 THz (1 GHz), the electromagnetic wave is able to excite the hot electrons and induce the plasmon oscillation in graphene.

**Figure 2:**
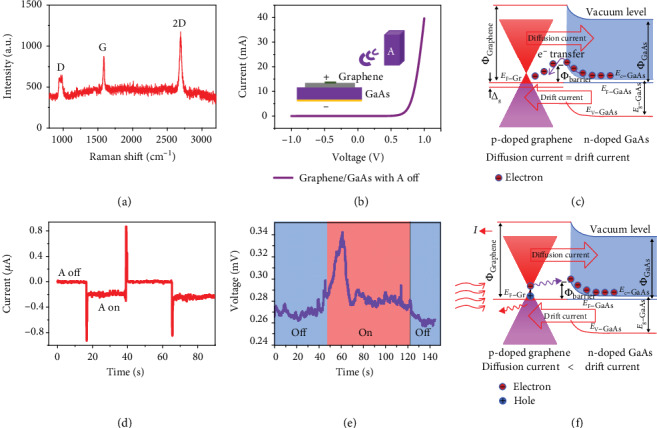
The performance characterization of the graphene/GaAs heterojunction energy harvester. (a) Raman spectrum of monolayer graphene. (b) Current versus voltage curve of graphene/GaAs with the RF source off. (c) Electronic band structure of independent graphene and GaAs. (d) Current characteristic versus time when switching the RF source off and on at a cycle of about 15 s on the graphene/GaAs wireless generator. (e) Voltage characteristic versus time when switching the RF source on and off on the graphene/GaAs wireless generator. (f) Schematic electronic band structure of the graphene/GaAs Schottky junction with the RF source on.

The schematic diagram of energy examination on the graphene/GaAs is shown in Figure 1(d), which consists of Ag electrode, monolayer graphene, n-doped GaAs, and Ti/Au back electrode.  Figure 2(a) displays the Raman spectrum of graphene and three peaks known as D peak, G peak, and 2D peak. The D peak around 964 cm^−1^ is very weak, showing that the graphene is of high quality. The G peak locates at around 1588 cm^−1^, which is blue-shifted comparing to 1580 cm^−1^ of intrinsic graphene, implying graphene is p-doped after the wet transferring process [23, 24].  Figure 2(b) depicts the dark current characteristic versus voltage of the graphene/GaAs Schottky diode at room temperature [23, 25], which indeed indicates that the contact between graphene and GaAs forms a rectifying diode and can be expressed as the following equation:

(2) $$\begin{array}{c} J=J_{\text{sT}}\left[\exp\left(\frac{eV}{{nk}_{\text{B}}T}\right)-1\right], \end{array}$$

where *e* is the value of the electron charge, *V* is the bias voltage, *k*_B_ is the Boltzmann constant, parameter *n* is called the ideality factor, and *J*_sT_ is the reverse saturation current density which is expressed by

(3) $$\begin{array}{c} J_{\text{sT}}=A^{*}T^{2}\exp\left(\frac{-e\phi_{\text{barrier}}}{k_{\text{B}}T}\right), \end{array}$$

where *A*^∗^ is the effective Richardson constant for thermionic emission [23, 26] and *Φ*_barrier_ can be deduced from equations (2) and (3).

The electronic band diagram of the graphene can be drawn and shown on the left of Figure 2(c) [27, 28]. Distinct from the traditional Schottky diode, the Fermi level of graphene can be shifted by external bias voltage and doping due to the low density of electronic states of graphene near the Dirac point. As a result, the static electron transfer between graphene and GaAs will change the Fermi level of graphene to some extent, which is expressed as Δ_g_ shown in Figure 2(c) [23, 27, 29, 30]. The schematic electronic band structure of the graphene/GaAs Schottky diode without RF illumination is shown in Figure 2(c). Since the GaAs is heavily n-doped herein, the conductance band (*E*_C−GaAs_) is close to its Fermi level (*E*_F−GaAs_). The electrons in the conductance of GaAs tend to transfer to graphene, which moves up the Fermi level of graphene, increases *Φ*_barrier_ of the junction, and consequently forms a balanced built-in electric field between graphene and GaAs [24]. In such a configuration, the drift current created by the built-in electric field is balanced with diffusion current formed by the diffusion of electrons. Thus, there is no current under dark condition without RF illumination. *Φ*_barrier_ can be written as

(4) $$\begin{array}{c} \phi_{\text{barrier}}=\phi_{\text{Graphene}}-\chi_{\text{GaAs}}-\Delta_{\text{g}}, \end{array}$$

where *χ*_GaAs_ is the electron affinity of GaAs, *Φ*_Graphene_ is the work function of graphene, and Δ_g_ is the shift of the Fermi level of graphene caused by the electron diffusion [24, 31]. The flowing direction of electrons is assumed as from graphene to semiconductor, which involves the tunneling process. On the other hand, if the electrons flow from semiconductor to graphene, it should also overcome the barrier height for tunneling; thus, we assume the electrons flow from graphene to semiconductor, which do not alter the main physical picture of our wireless graphene/semiconductor energy harvester.

All experiments were conducted in dark conditions shown in Figure 1(d). As illustrated in Figure 2(d), the dark current is in the order of 10^−9^ A at zero bias without 915 MHz RF emission. However, when putting the harvester under the RF illumination, the current increases rapidly more than one order of magnitude and the value can reach in the order of 10^−8^–10^−7^ A, which indicates energy generation exited by RF emission.  Figure 2(e) shows the voltage of the graphene/GaAs Schottky diode with the RF source on and off. When the RF source is on, the difference of voltage can reach 0.08 mV, which, however, is not as stable and direct as current as a result of the quantum nature of the incident electromagnetic wave herein. These phenomena indeed show that the graphene/GaAs diode harvests the 915 MHz RF waves and the contact between graphene and GaAs can effectively convert incident RF waves into electricity, indicating the transfer of carriers internally. We also conducted the experiment of graphene/GaN heterojunction which shows different current outputs, possibly due to the larger barrier height of the graphene/GaN heterostructure compared with the graphene/GaAs heterostructure (supporting information). According to the experimental results and the plasmon theory proposed before, we get the schematic electronic band structure of the graphene/GaAs heterostructure in Figure 2(f) which shows the dynamic carrier transfer process of the device. When putting the sample under the 915 MHz source, the plasmon is stimulated near the Dirac point of graphene and abundant hot hole-electron pairs appear, and electrons in the conductance band of graphene tend to tunnel through the barrier into the GaAs at a rapid rate due to the built-in electric field. Thus, the balance between built-in electric filed and diffusion electrons is destroyed [32]. It is intriguing that there are obvious current spikes when we turn on and off the RF emission source as shown in Figure 2(d). This abrupt current peak could be induced by the unstable and strong RF emission when we open or turn off the RF source.

**Figure 3:**
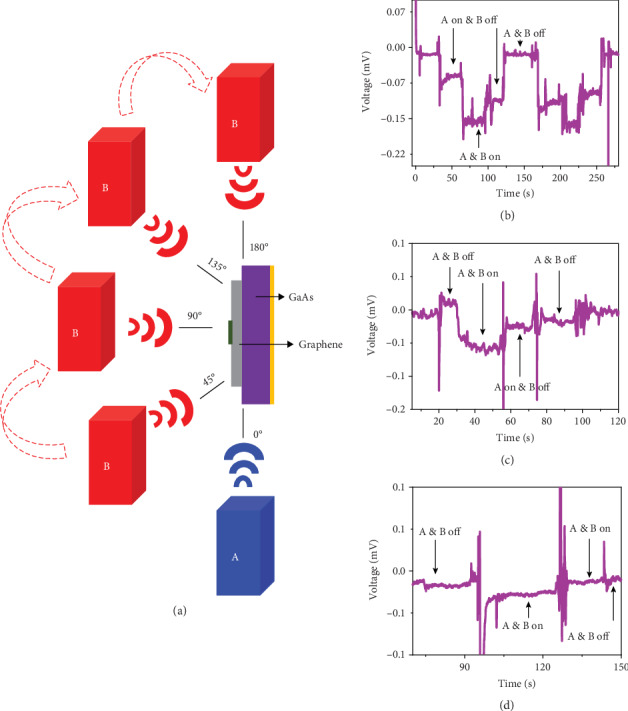
The performance characterization of the graphene/GaAs heterojunction energy harvester with two wireless energy sources. (a) The relative position of emissions and device. (b) Voltage characteristic versus time of the device when switching the RF sources off and on forming an angle of 45° on the graphene/GaAs wireless generator. (c) Voltage characteristic versus time when switching the RF sources off and on forming an angle of 90° on the graphene/GaAs wireless generator. (d) Voltage characteristic versus time when switching the RF sources off and on forming an angle of 180° on the graphene/GaAs wireless generator.

We also conducted the experiment under three sources to check whether the DC power caused by different sources will superpose. We placed the device as the center of a circle and two sources at the circle of the radius of 15 cm, forming some different angles as Figure 3(a) shows, and the results are illustrated in Figures 3(b)–3(d). It is found that the current generated by the superposition of two sources is larger than any of them when the angle is smaller (≥0°), but it is not strictly equal to the sum of the currents generated by two sources. With the increase of the angle, the superimposed current is less than that generated by a single source, and this effect is most obvious when the angle is increased to 180°. Turning on the second source will lead to the reduction of the current obtained by turning on the first source alone. From the experimental data, we suppose there is a correlation between the data and the wave characteristics. It is speculated that the result of multisource superposition is similar to that of wave superposition, which still needs further experimental verification.

**Figure 4:**
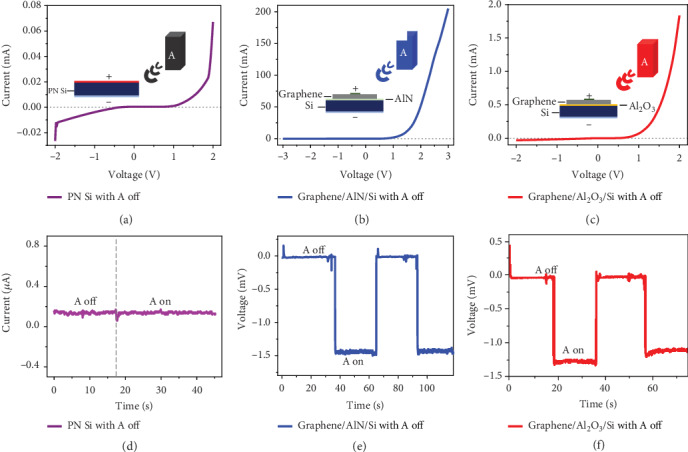
The performance characterization of the graphene/semiconductor heterostructure wireless energy harvester. (a)I-V characteristic of PN Si with the RF source off. (b) I-V characteristic of graphene/AlN/Si with the RF source off. (c) I-V characteristic of graphene/Al_2_O_3_/Si with the RF source off. (d) The current variation of the Si wireless generator when switching the RF source off and on. (e) The current variation of the graphene/AlN/Si wireless generator when switching the RF source off and on. (f) The current variation of the graphene/Al_2_O_3_/Si wireless generator when switching the RF source off and on.

To further enhance the harvesting efficiency, the device can be fabricated using integrated heterostructure array, which can reduce the parasitic capacitances between graphene and substrate by decreasing the contact area. Instead of a large area, using some small areas in integrated heterostructure array can improve the current density and make the device more solid. Besides, we can adopt the method of inserting an insulating layer between graphene and semiconductor such as GaAs and Si to get higher current density by reducing the charge-carrier recombination. We conducted the experiment of inserting AlN or Al_2_O_3_ between graphene and Si as the insulating layer for carrier transport to obtain better performance of the wireless energy generator. Since the silicon is sensitive to THz, we firstly detect the current it generated with RF emission on and off in order to test whether the silicon is sensitive to 915 MHz or not, which is illustrated in Figure 4(d), indicating that Si alone cannot generate electricity under 915 MHz RF illumination. And the dark I-V curve of Si which is processed as a diode is illustrated in Figure 4(a). Figures 4(b) and 4(c) show dark I-V curves of graphene/AlN/Si and graphene/Al_2_O_3_/Si just like the dark I-V curve of graphene/GaAs mentioned before, which indicates that they are indeed rectifying diodes. AlN and Al_2_O_3_ are all insulating layers used for carrier transport layers, which can improve carrier transport efficiency so that the graphene/Si Schottky diode can be optimized. Therefore, there is higher current generated by graphene/AlN/Si and graphene/Al_2_O_3_/Si comparing to graphene/GaAs. It is found that the current of graphene/AlN/Si is about 0.25 *μ*A and the current of graphene/Al_2_O_3_/Si is 0.2 *μ*A. As shown in Figures 4(e) and 4(f), the voltage of the graphene/AlN/Si diode can reach about 1.5 mV and the voltage of the graphene/Al_2_O_3_/Si diode can reach as high as 1.3 mV when the RF source is open. Comparing to the voltage that graphene/GaAs generates, graphene/AlN/Si and graphene/Al_2_O_3_/Si can greatly optimize the wireless energy generator, which can be seen that the voltage is stable and the value is enhanced. Considering the current generated by electromagnetic wave with different wavelengths, we predict that waves with shorter wave length will harvest larger current. As mentioned before, electrons are excited when photons are significantly greater than bandgap energy. So, the internal carrier emission efficiency is raised when the wavelength gets shorter, thus high frequency and high energy according to the photon energy formula. In this case, it also shows promising application scenarios of the wavelength detector for the wireless source.

## 3. Conclusion

In summary, we have demonstrated the feasibility of harvesting wireless energy around 1 GHz through a simple graphene/semiconductor heterostructure, which utilizes the unique physical properties of hot electrons of graphene and the ultrafast carrier dynamics between graphene and semiconductor. Moreover, graphene is active for higher frequency wireless energy reaching the scale of THz energy. Therefore, the utilization of the millimeter-wave spectrum to provide power will be one of the most promising ways to support the long-term running of the wireless sensors.

## Supplementary Materials

Supplementary materials Figure S1: time dependence of current on graphene/GaAs. Figure S2: time dependence of current and voltage on graphene/GaN. Figure S3: the performance characterization of the graphene/GaAs heterojunction energy harvester with two wireless energy sources.
