# Supplementary material for: Graphene/Semiconductor Heterostructure Wireless Energy Harvester through Hot Electron Excitation
Source: Research (Wash D C). 2020 Jun 8;2020:3850389. doi: 10.34133/2020/3850389 (PMC7298352; doi:10.34133/2020/3850389)
Supplement: Supplementary materials — Figure S1: time dependence of current on graphene/GaAs. Figure S2: time dependence of current and voltage on graphene/GaN. Figure S3: the performance characterization of the graphene/GaAs heterojunction energy harvester with two wireless energy sources. [file 3850389.f1.zip › 3850389.f1/Supporting .docx]

**Type: Research Article**

**Graphene/semiconductor heterostructure wireless energy harvester through hot electron excitation**

*Yangfan Xuan^1,&^, Hong Chen^1,&^, Yan Chen^1,&^, Yanghua Lu^1^, Haonan Zheng^1^, Shisheng Lin^1,2,*^*

^1^College of microelectronics, College of Information Science and Electronic Engineering, Zhejiang University, Hangzhou, 310027, P. R. China

^2^State Key Laboratory of Modern Optical Instrumentation, Zhejiang University, Hangzhou, 310027, P. R. China

*Correspondence: [shishenglin@zju.edu.cn](mailto:shishenglin@zju.edu.cn).

Supplementary Figures:

**1. Figure S1**


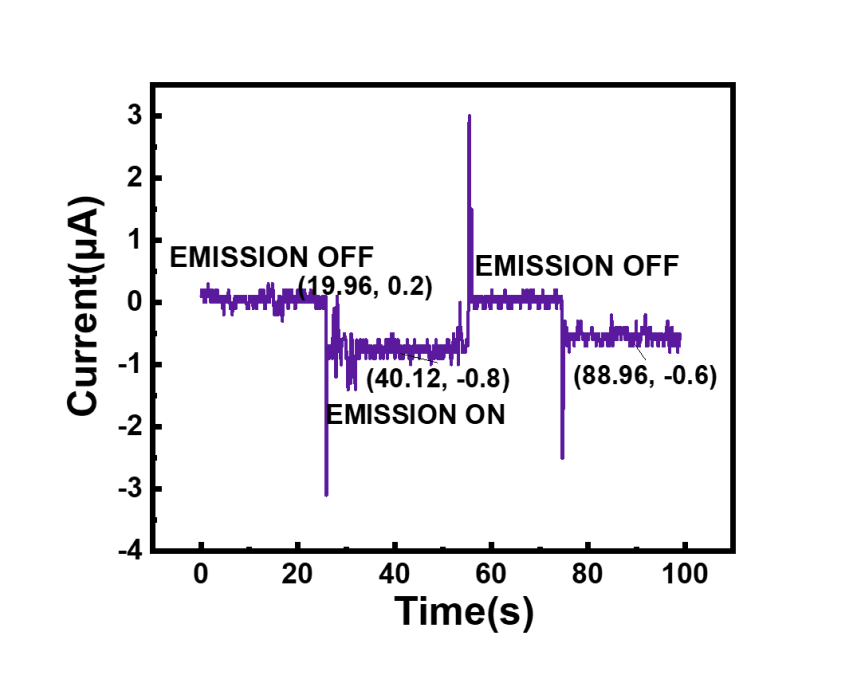


**Figure S1:** **Time dependence of current on graphene/GaAs.** Time dependence of current when switching RF source off and on at a cycle of about 20s on graphene/GaAs wireless generator.

**2. Figure S2**


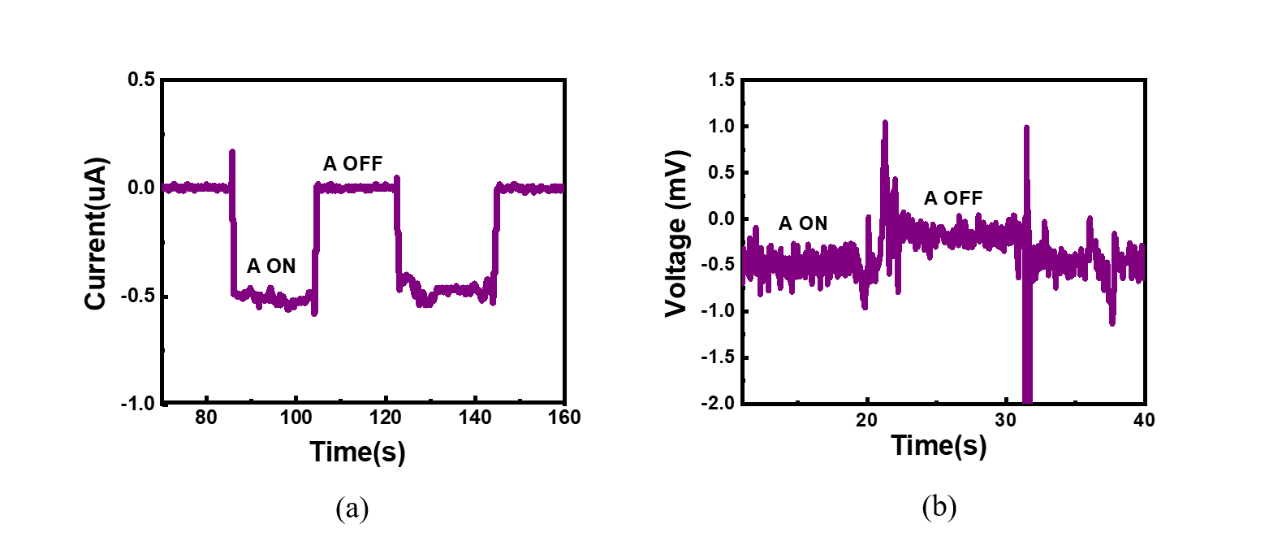


**Figure S2: Time dependence of current and voltage** **on graphene/GaN.** (a) Time dependence of current when switching RF source off and on at a cycle of about 20s on graphene/GaN wireless generator. (b) Time dependence of voltage when switching RF source off and on at a cycle of about 20s on graphene/GaN wireless generator.

**3. Figure S3**


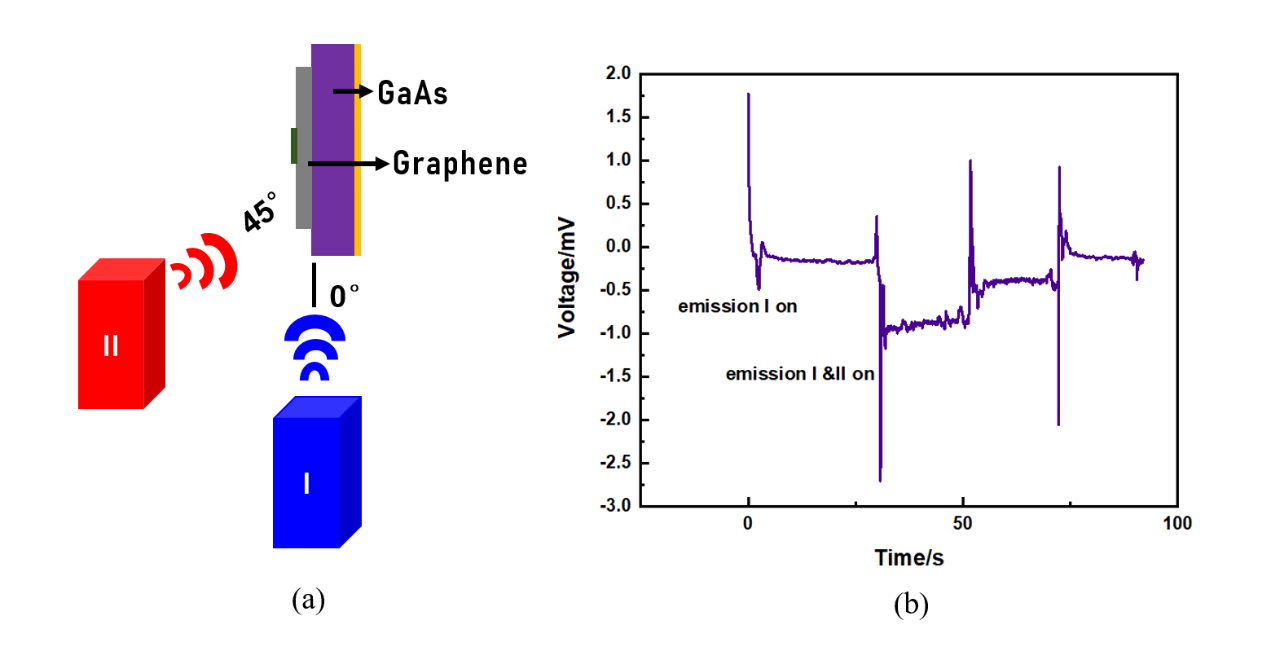


**Figure S3: The** **performance characterization of the graphene/GaAs heterojunction energy harvester with two wireless energy sources.** (a) The relative position of emissions and device. (b) Time dependence of current when switching two sources which form an angle of 45°.
