# Supplementary figures and images for: Graphene/Semiconductor Heterostructure Wireless Energy Harvester through Hot Electron Excitation
Source: Research (Wash D C). 2020 Jun 8;2020:3850389. doi: 10.34133/2020/3850389 (PMC7298352; doi:10.34133/2020/3850389)

## Slide 1
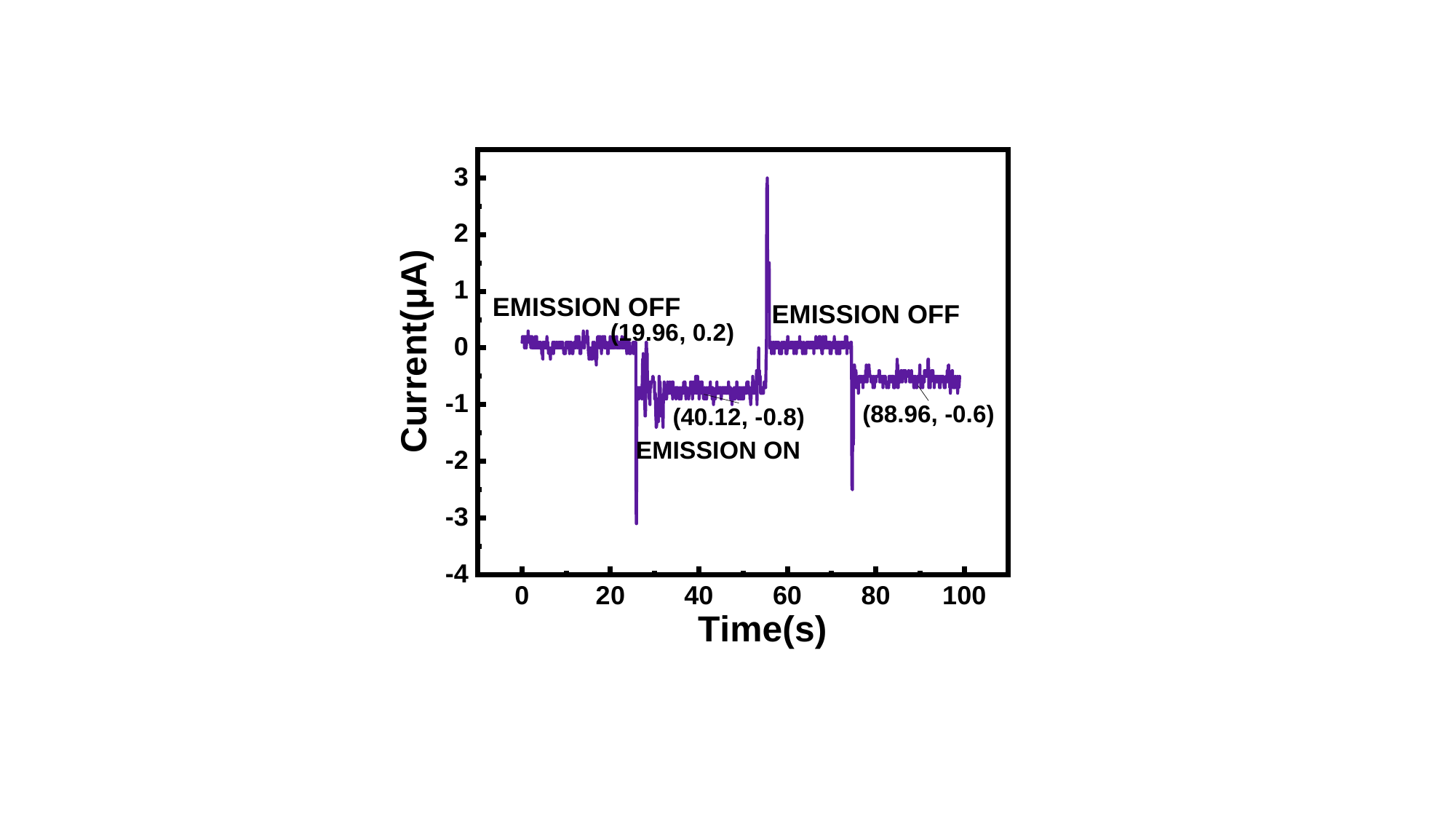

Supplement: Supplementary materials — Figure S1: time dependence of current on graphene/GaAs. Figure S2: time dependence of current and voltage on graphene/GaN. Figure S3: the performance characterization of the graphene/GaAs heterojunction energy harvester with two wireless energy sources. [file 3850389.f1.zip › 3850389.f1/Supporting-fig1.pptx]

## Slide 1
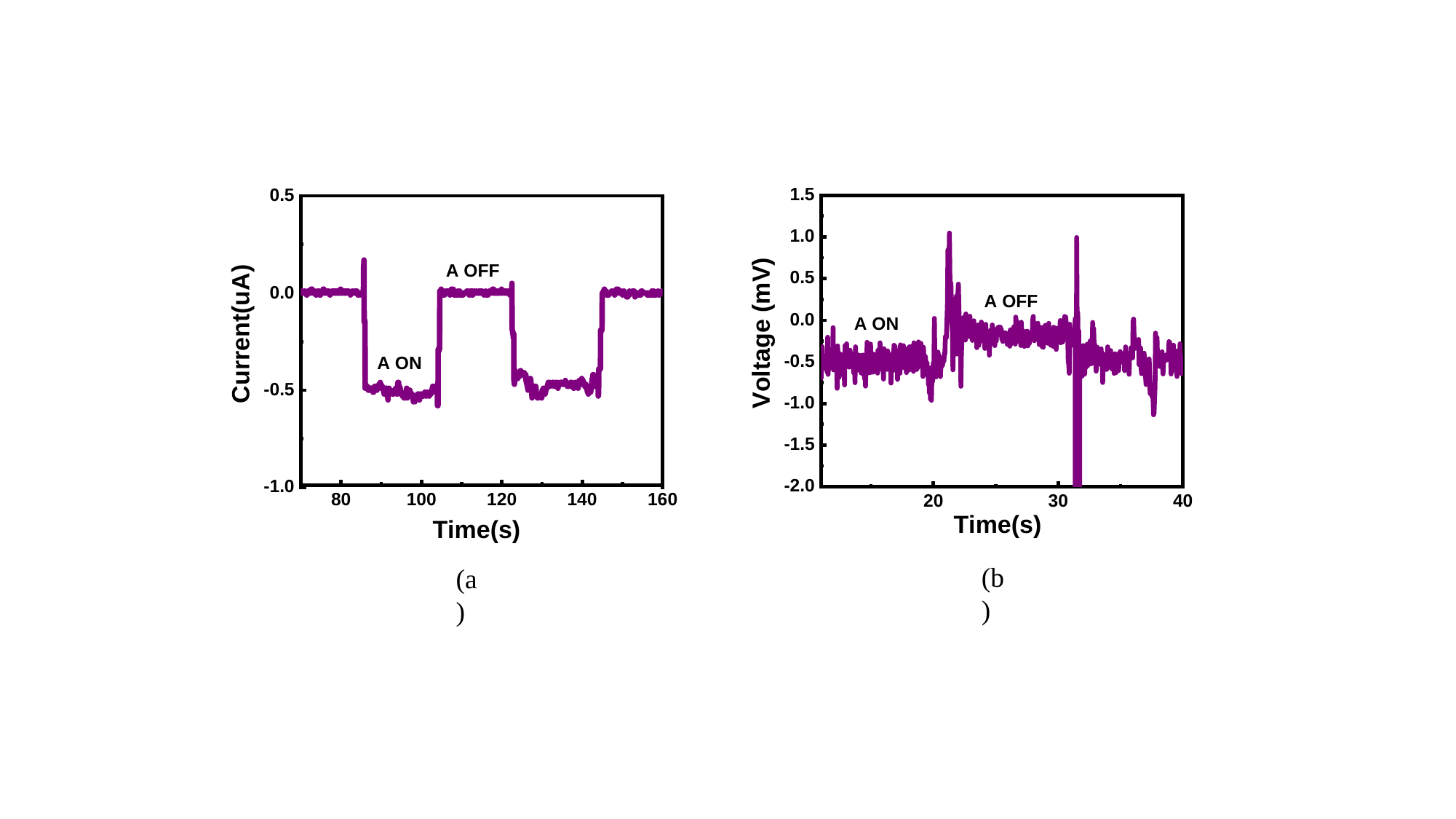

(b)
(a)

Supplement: Supplementary materials — Figure S1: time dependence of current on graphene/GaAs. Figure S2: time dependence of current and voltage on graphene/GaN. Figure S3: the performance characterization of the graphene/GaAs heterojunction energy harvester with two wireless energy sources. [file 3850389.f1.zip › 3850389.f1/Supporting-fig2.pptx]

## Slide 1
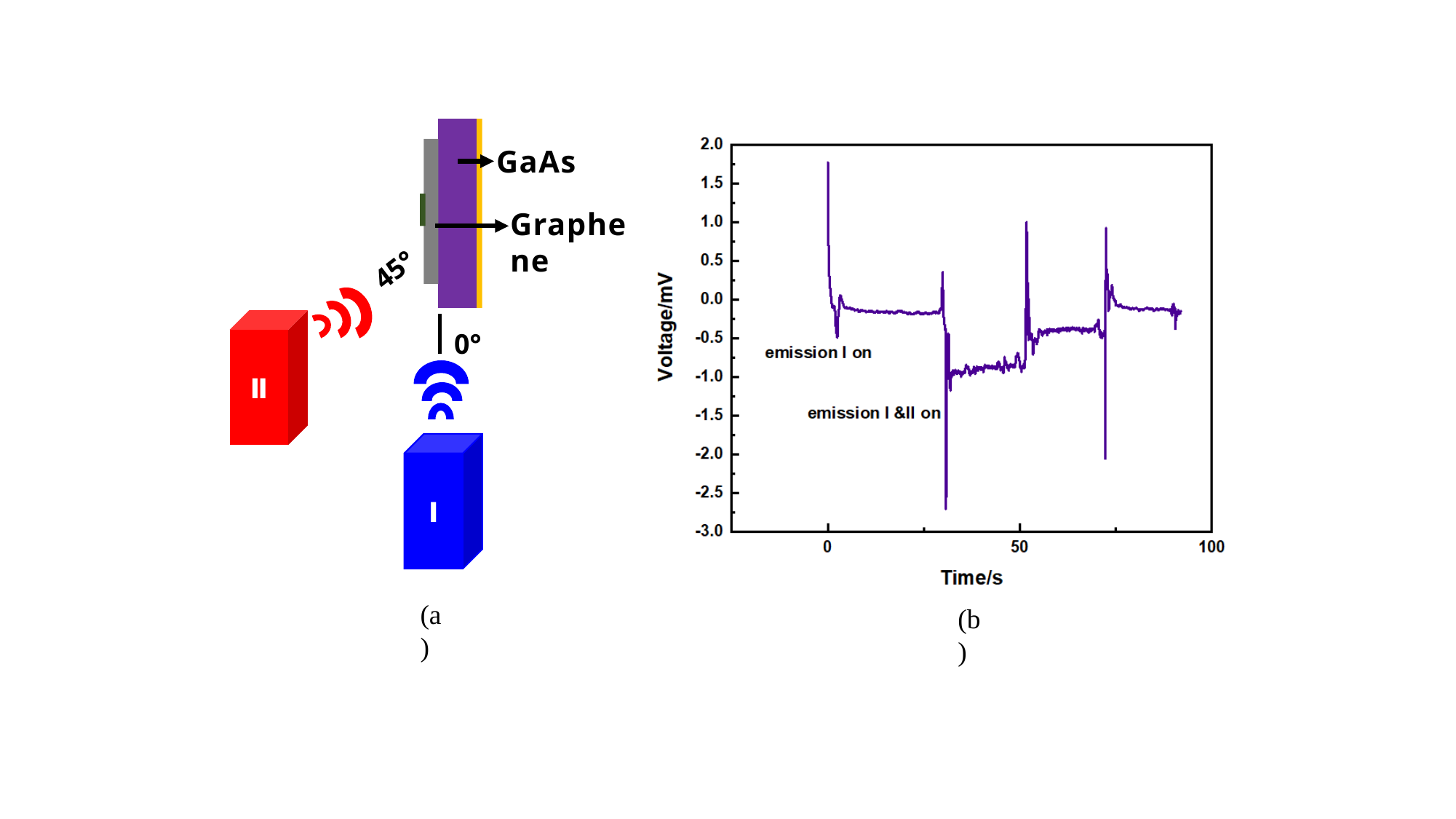

GaAs
Graphene
45°
Ⅱ
0°
Ⅰ
(a)
(b)

Supplement: Supplementary materials — Figure S1: time dependence of current on graphene/GaAs. Figure S2: time dependence of current and voltage on graphene/GaN. Figure S3: the performance characterization of the graphene/GaAs heterojunction energy harvester with two wireless energy sources. [file 3850389.f1.zip › 3850389.f1/Supporting-fig3.pptx]
